# The Relationship between Brown Adipose Tissue Activity and Neoplastic Status: an ^18^F-FDG PET/CT Study in the Tropics

**DOI:** 10.1186/1476-511X-10-238

**Published:** 2011-12-20

**Authors:** Yung-Cheng Huang, Tai-Been Chen, Chien-Chin Hsu, Shau-Hsuan Li, Pei-Wen Wang, Bi-Fang Lee, Ching-Yuan Kuo, Nan-Tsing Chiu

**Affiliations:** 1 Department of Nuclear Medicine, Kaohsiung Chang Gung Memorial Hospital and Chang Gung University College of Medicine, Kaohsiung, Taiwan; 2 Department of Information Engineering, I-Shou University, Kaohsiung, Taiwan; 3 Department of Medical Imaging and Radiological Sciences, I-Shou University, Kaohsiung, Taiwan; 4 Divisions of Hemato-Oncology, Department of Internal Medicine, Kaohsiung Chang Gung Memorial Hospital and Chang Gung University College of Medicine, Kaohsiung, Taiwan; 5 Department of Nuclear Medicine, College of Medicine and Hospital, National Cheng Kung University, Tainan, Taiwan

**Keywords:** Neoplastic status, BAT, ^18^F-FDG PET

## Abstract

**Background:**

Brown adipose tissue (BAT) has thermogenic potential. For its activation, cold exposure is considered a critical factor though other determinants have also been reported. The purpose of this study was to assess the relationship between neoplastic status and BAT activity by 2-deoxy-2-[18F]fluoro-D-glucose (^18^F-FDG) positron emission tomography/computed tomography (PET/CT) in people living in the tropics, where the influence of outdoor temperature was low.

**Methods:**

^18^F-FDG PET/CT scans were reviewed and the total metabolic activity (TMA) of identified activated BAT quantified. The distribution and TMA of activated BAT were compared between patients with and without a cancer history. The neoplastic status of patients was scored according to their cancer history and ^18^F-FDG PET/CT findings. We evaluated the relationships between the TMA of BAT and neoplastic status along with other factors: age, body mass index, fasting blood sugar, gender, and outdoor temperature.

**Results:**

Thirty of 1740 patients had activated BAT. Those with a cancer history had wider BAT distribution (*p *= 0.043) and a higher TMA (*p *= 0.028) than those without. A higher neoplastic status score was associated with a higher average TMA. Multivariate analyses showed that neoplastic status was the only factor significantly associated with the TMA of activated BAT (*p *= 0.016).

**Conclusions:**

Neoplastic status is a critical determinant of BAT activity in patients living in the tropics. More active neoplastic status was associated with more vigorous TMA of BAT.

## Background

Brown adipose tissue (BAT) is a highly specialized thermogenic tissue that is essential for non-shivering thermogenesis [1]. BAT activation helps maintain normal body temperatures in newborns. Although the amount of BAT declines with age, islets of brown adipocytes remain in the white adipose tissue of adult humans [1,2]. High levels of BAT in the adult body are associated with cancer-induced cachexia and may reflect an abnormal mechanism responsible for substantial energy expenditure and subsequent weight loss [3]. BAT has recently attracted attention because it consumes stored energy and may thereby be involved in human obesity and age-related metabolic diseases [4,5]. The two extremes in the regulation of body weight, obesity and cachexia can be considered two sides of the same coin [6,7]. Beyond the well-known influence of outdoor temperature on activated BAT, little has been published on the impact of neoplastic status. We did this study to evaluate the relationship between BAT activity and neoplastic status by 2-deoxy-2-[18F]fluoro-D-glucose (^18^F-FDG) positron emission tomography/computed tomography (PET/CT) in people living in the tropics, where the influence of outdoor temperature is low.

## Methods

### Patients

Between June 2005 and May 2009, 1740 patients at our institution (located at 22.7°N) underwent 1903 consecutive clinical ^18^F-FDG PET/CT scans for a variety of purposes; these scans were reviewed for analysis. This retrospective study was approved by our hospital's Institutional Review Board with a waiver of consent.

### ^18^F-FDG PET/CT

^18^F-FDG PET/CT has been successfully used to diagnose various cancers and inflammatory processes [8] as well as to visualize functional activated BAT [9-13] based on the presence of uncoupling protein 1 (UCP1)-positive adipocytes with multilobulated lipid droplets [14]. To identify activated BAT, we used an in-line PET/CT system that could clearly localize the ^18^F-FDG uptake to fat tissue and exclude muscle and lymph nodes. To estimate the total metabolic activity (TMA) of BAT, we used three-dimensional (3D) voxel-based analysis rather than the two-dimensional (2D) region of interest method, taking both volume and ^18^F-FDG intensity into consideration, which is an accurate method for quantifying activated BAT in the living human body.

PET/CT scans were done using a combined PET/CT scanner (Discovery ST; GE Healthcare, Waukesha, WI, USA). The patients fasted for at least 6 hours before the ^18^F-FDG injection. No attempt was made to prevent BAT activation before the PET/CT scan by prescribing any medication such as beta-blockers or diazepam. The patients were injected with 370-555 MBq of ^18^F-FDG and the dose were adjusted according to body weight in pediatric patients. Then, they remained recumbent in an isolated, continually air-conditioned and dimly lit room for 1 hour. CT images were acquired first without contrast medium using the following parameters: 140 kV, 170 mA (maximum), and 3.75-mm thick sections for attenuation correction and the later imaging fusion. PET scans were then taken from the mid-thigh to skull with 5-7 bed positions of 5 minutes each. The transaxial PET data were reconstructed as 128 × 128-pixel images with a slice thickness of 3.27 mm, a spatial resolution of 6.2-6.9 mm full width at half-maximum (FWHM), and an axial resolution of 4.6-6.1 mm FWHM by applying an ordered subsets expectation maximization algorithm (OSEM, 2 iterations, 30 subsets). Coronal, and sagittal sections, as well as maximum intensity projection PET images, were also reformatted for PET/CT imaging fusion and interpretation.

### Identifying brown adipose tissue and measuring its total metabolic activity

**Figure 1:**
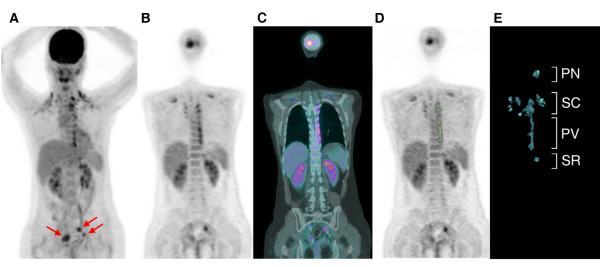
**18F-FDG PET/CT scan of a 33-year-old woman with a history of cervical cancer treated with a radical hysterectomy: (A) maximum intensity projection, (B) coronal PET, and (C) coronal PET/CT fusion images showing activated BAT deposits in the posterior neck (PN), supraclavicular (SC), paravertebral (PV) and suprarenal (SR) areas**. The hypermetabolic foci in the pelvis were locally recurrent tumors (red arrows) and the neoplastic status score was 3. (D) We used OsiriX 64-bit software with the seeded region growing method (green zone) to obtain the volume of interest (VOI) of activated BAT from the PET images. (E) Color image segmentation based on 3-dimensional clustering was used for display and volume computation. Total metabolic activity (TMA) was defined as the summation of the volume × standardized uptake value (SUV) within the VOIs of the activated BAT deposits.

^18^F-FDG PET/CT scans with reports stating that the patients had activated BAT were reviewed again by two experienced nuclear medicine physicians to confirm the presence of activated BAT and identify its anatomical distribution. Activated BAT was considered present if there were areas of ^18^F-FDG uptake corresponding to the CT density of adipose tissue (-250 to -50 Hounsfield units) that were distinguishable from nearby tissue by distinct patterns consistent with BAT [11,15]. The anatomical distribution of increased ^18^F-FDG uptake corresponding to BAT on fused PET/CT images was classified as being in the posterior neck (PN), supraclavicular (SC) including mediastinal extension, paravertebral (PV), or suprarenal (SR) regions (Figure 1). The distribution of activated BAT, age, body mass index (BMI), fasting blood sugar, and gender of patients with activated BAT were recorded.

For 3D voxel-based analysis, the volume and ^18^F-FDG uptake of each activated BAT deposit were measured using OsiriX 64-bit software (version 3.7.1; Antoine Rosset; http://www.osirix-viewer.com/index.html) run on a Macintosh OS × platform. The standardized uptake value (SUV) within the region of interest was calculated semi-quantitatively according to the formula:

$$\text{SUV}=\text{measured}\;\text{activity}\left(\text{MBq}∕\text{mL}\right)∕\left[\text{injected}\;\text{does}\;\text{o}{\text{f}}^{18}\text{F - FDG}\left(\text{MBq}\right)∕\text{body}\;\text{weight}\left(\text{g}\right)\right]$$

The seeded region growing method with the lower bound set initially at an SUV of 2.5 was used to determine the volume of interest (VOI) of the activated BAT. The analysis required agreement between the two physicians, and the results confirmed by the fused CT images. A maximum adjustment of 0.5 for the lower bound of the SUV was allowed to better define the VOI and adjust for the effects of unsatisfactory contrast between the BAT and background activity (Figures 1D and 1E). The TMA was defined as the summation of the volume × SUV within the VOIs of each patient's total activated BAT deposits. Only the first PET/CT study of each patient was included for analysis of continuous variables.

### Determining neoplastic status

The neoplastic status of patients with activated BAT was scored according to their cancer history and ^18^F-FDG PET/CT findings as follows: 0, patients who underwent PET/CT for cancer screening without a history of cancer and had negative findings on the PET/CT scan; 1, patients who had a history of cancer but had negative findings on the PET/CT scan; 2, patients with a history of cancer and on the PET/CT scan had abnormal uptake probably related to inflammation or malignancy but who were lost to follow-up before cancer could be confirmed by pathology or additional imaging studies; and 3, patients who on the PET/CT scan had pathological uptake because of cancer confirmed by pathology or follow-up imaging.

### Outdoor temperature parameters

For our patients with activated BAT, the daily average outdoor temperatures in °C for the date of the PET study (T_PET_) and the 7 days prior (T_1-7_) were recorded. The 1-day temperature difference (T_PET _- T_1_), mean daily average temperature [mean T = (T_PET _+ T_1 _+... T_7_)/8], and maximum continuous temperature decrease within the 7 days before the PET study (maxΔT) in °C were calculated. The temperature descent grade (DG = maxΔT/mean T) and descent rate (DR = maxΔT/days of continuous temperature descent) were also calculated. The outdoor daily average temperature data were obtained from the Central Weather Bureau of Taiwan.

### Statistical analysis

Continuous variables are means ± standard error (SE). Kolmogorov-Smirnov statistics were used to test the data sets for normal distribution. Student's *t-*test was used in group comparisons of normally distributed data, and the Mann-Whitney *U-*test was used for non-normally distributed data. Categorical variables were analyzed using the χ^2 ^test. For patients with activated BAT, univariate analyses consisted of Pearson's correlations, Spearman's rank correlation, and Mann-Whitney *U*-test of the associations between the TMA of BAT and biological and environmental factors of age, BMI, fasting blood sugar, gender, and outdoor temperature parameters. The TMA of BAT in the 4 different neoplastic status categories was analyzed using the Jonckheere-Terpstra test for ordered alternatives, a nonparametric test for trends. Multiple linear regression analysis was used to test the significance of the associations between the TMA of BAT and the biological and environmental factors. SPSS 17 for Windows (SPSS Inc., Chicago, IL, USA) was used for all statistical analysis. Significance was set at *p *< 0.05.

## Results

Thirty (26 females and 4 males; age range: 12-73 years old; mean age: 40.6 ± 2.7 years old) of the 1740 patients had activated BAT, which was identified on 37 scans. The demographic features of the patients are summarized in Table 1. There were 1,171 patients with a history of cancer and were given an ^18^F-FDG PET/CT scan to evaluate the disease. The other 569 patients without a history of cancer requested an ^18^F-FDG PET/CT scan for a cancer survey. The prevalence of activated BAT in the two groups was not significantly different: 1.79% and 1.58%, respectively. However, patients with activated BAT were significantly younger, leaner, more often female, had lower fasting blood sugar, and underwent an ^18^F-FDG PET/CT scan during lower outdoor temperature conditions than did the patients without activated BAT.

**Table 1:** Demographic features of patients with and without activated BAT detected using ^18^F-FDG PET/CT

Characteristic	With activated BAT (n = 30)	Without activated BAT (n = 1710)	*p-*value
Age (y)	40.6 ± 2.7 (12-73)	53.8 ± 0.3 (1-86)	< 0.001*
BMI (kg/m^2^)	21.5 ± 0.6 (16.4-32.9)	24.0 ± 0.1 (13.0-40.6)	< 0.001*
Fasting blood sugar (mg/dL)	83.6 ± 1.8 (56-104)	91.4 ± 0.4 (52-192)	0.032*
Gender (female:male)	26:4	697:1013	< 0.001^†^
Outdoor temperature (°C)	23.0 ± 0.7(16.7-29.6)	25.3 ± 0.1 (14.2-31.5)	0.001^‡^
Cancer: Non-cancer	21:9	1150: 560	0.750^†^

Data are means ± standard error and (range).

*Student's *t-*test; ^†^χ^2 ^test; ^‡^Mann-Whitney *U-*test.

**Figure 2:**
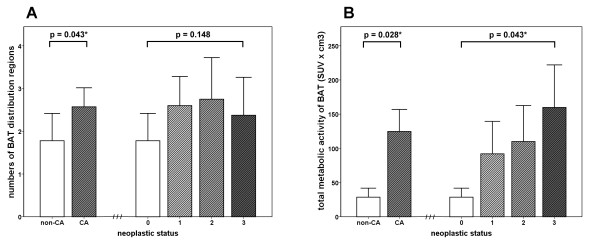
**The association between BAT activity and neoplastic status**. (A) BAT distribution regions and (B) total metabolic activity (TMA) of the BAT deposits in patients with different neoplastic status. The distribution of activated BAT was significantly (*p *= 0.043) wider in patients with a history of cancer than in those without. The TMA of activated BAT was significantly (*p *= 0.028) higher in patients with a history of cancer than in those without. A higher neoplastic status score was associated with a higher average TMA of BAT (Jonckheere-Terpstra Trend Test across the 4 groups, *p *= 0.043), although there was a positive but non-significant association between neoplastic status and distribution of BAT (*p *= 0.148). Error bars indicate standard error of the mean.

**Figure 3:**
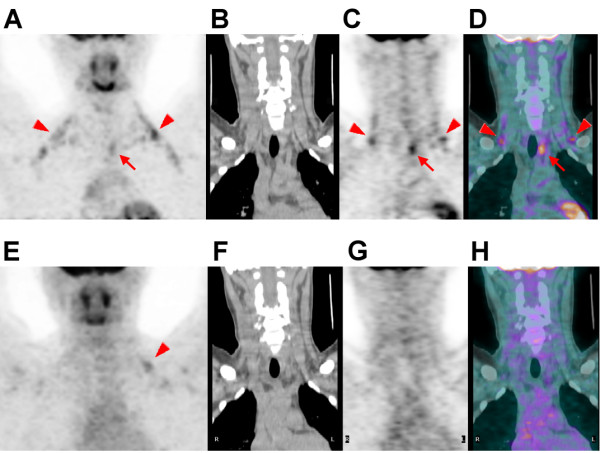
**^18^F-FDG PET/CT scan (top row) of an 18-year-old man with a history of papillary thyroid cancer treated with a bilateral total thyroidectomy showing activated BAT deposits in the bilateral posterior neck (PN) and supraclavicular (SC) areas (arrowheads), and a hypermetabolic nodule in the left paratracheal area (arrows)**. The nodule was surgically excised and proved to be a metastatic lymph node. At that time, the TMA of the BAT was 46.6, the neoplastic status score was 3, the outdoor daily average temperature on the date of the PET/CT study was 25.4 °C, the patient's BMI was 29.0 kg/m^2^, and his fasting blood sugar was 86 mg/dL. On the follow-up ^18^F-FDG PET/CT scan (bottom row) after the lymph node had been surgically excised, there was no abnormal tumor uptake and only minimal activated BAT deposits in the SC areas (E, arrowhead). At that time, the TMA of the BAT was 9.2, the neoplastic status score was 1, the outdoor daily average temperature on the date of the PET/CT study was 24.4 °C, the patient's BMI was 28.1 kg/m^2^, and his fasting blood sugar was 99 mg/dL. (A, E: maximum intensity projections; B, F: coronal CT images; C, G: coronal PET images; D, H: coronal PET/CT fusion images).

Most activated BAT was found in the supraclavicular area, and the least was found in the suprarenal area. Activated BAT was found in only one distribution region in 8 patients, 2 regions in 7 patients, 3 regions in 12 patients, and 4 regions in 3 patients. The imaging and demographic characteristics of patients with activated BAT on ^18^F-FDG PET/CT are summarized in Tables 2 and 3. The distribution of activated BAT was significant wider in patients with a history of cancer than without (*p *= 0.043). The TMA of activated BAT was higher in patients with a history of cancer than without (*p *= 0.028). A higher neoplastic status score was associated with a higher average TMA of BAT (Jonckheere-Terpstra Trend Test across the 4 groups, *p *= 0.043), although there was a positive but non-significant association between neoplastic status and the distribution of BAT (*p *= 0.148) (Figure 2). In univariate analysis, no significant association was found between the TMA of BAT and age, BMI, fasting blood sugar, gender, or outdoor temperature parameters. Only the neoplastic status was significantly associated with the TMA of BAT. A multiple linear regression analysis showed that neoplastic status remained the only significant determinant of the TMA of BAT (*p *= 0.016) (Table 4). We also found one patient with repeated ^18^F-FDG PET scans who showed lower BAT metabolic activity after surgical excision of his malignancy (Figure 3).

**Table 2:** Imaging characteristics of activated BAT on ^18^F-FDG PET/CT

Characteristic	*n*	Measured value
Anatomical distribution		
Posterior neck (SUV_max_)	21	3.5 ± 0.3 (1.6-6.6)
Supraclavicular (SUV_max_)	29	5.0 ± 0.5 (1.8-9.5)
Paravertebral (SUV_max_)	17	4.4 ± 0.5 (2.0-8.1)
Suprarenal (SUV_max_)	3	5.8 ± 0.7 (4.8-7.2)
BAT total metabolic volume (cm^3^)		32.1 ± 7.5 (0.1-143.5)
BAT total metabolic activity		95.9 ± 24.1 (0.2-467.5)
1 distribution region	8	4.7 ± 4.2 (0.2-34.4)
2 distribution regions	7	46.9 ± 20.0 (0.2-137.2)
3 distribution regions	12	131.0 ± 35.9 (13.2-423.7)
4 distribution regions	3	312.5 ± 114.4 (89.3-467.5)

Data are means ± standard error and (range).

SUV_max _: maximum standardized uptake value

**Table 3:** Univariate analysis of relationships between the total metabolic activity (TMA) of BAT and various biological and environmental factors (N = 30)

Factor	TMA	Correlation coefficient	*p-*value
NEOPLASTIC STATUS SCORE			0.043*
0 (n = 9)	28.6 ± 13.2 (0.2-91.3)		
1 (n = 5)	92.0 ± 47.4 (4.6-209.2)		
2 (n = 8)	110.0 ± 52.7 (0.3-467.5)		
3 (n = 8)	159.8 ± 62.2 (0.2-423.7)		
Age (y)		0.084	0.658^†^
BMI (kg/m^2^)		0.033	0.861^†^
Fasting blood sugar (mg/dL)		0.112	0.554^†^
GENDER			0.428^‡^
Male (n = 4)	149.0 ± 96.1 (0.2-423.7)		
Female (n = 26)	87.7 ± 24.2 (0.2-467.5)		
OUTDOOR TEMPERATURE PARAMETERS (°C)			
Outdoor temperature (T_PET_)		0.101	0.596^§^
MaxΔT		-0.297	0.110^§^
One-day temperature decline (T_PET _- T_1_)		0.171	0.366^§^
Mean T: (T_PET _+ T_1 _+... T_7_)/8		0.087	0.649^§^
Decline grade (maxΔT/mean T)		-0.254	0.181^§^
Decline rate (maxΔT/duration)		-0.319	0.086^§^

Data are means ± standard error and (range).

*Jonckheere-Terpstra test; ^†^Pearson's correlation; ^‡^Mann-Whitney *U-*test; ^§^Spearman's rank correlation.

**Table 4:** Multiple linear regression analysis of the relationships between total metabolic activity (TMA) of BAT and various biological and environmental factors (N = 30)

Factor	Coefficient	Standard error	*p-*value
Neoplastic status	51.377	19.686	0.016*
Age	3.304	1.842	0.086
BMI (kg/m^2^)	-10.508	9.392	0.275
Fasting blood sugar (mg/dL)	0.149	2.251	0.948
Gender (male)	135.308	102.922	0.202
Outdoor temperature T_PET _(°C)	10.178	5.903	0.098

* Statistically significant.

## Discussion

To explore the relationship between BAT activity and neoplastic status, we used ^18^F-FDG PET/CT to determine the distribution and TMA of activated BAT in our patients living in the tropics, where the influence of outdoor temperature was low and the effect of neoplastic status on the activation of BAT could be more clearly determined. We found that neoplastic status was a critical determinant of BAT activity.

In limited numbers of patients, a high prevalence of activated BAT has been reported in patients with lymphoma (17%) and in female patients with breast cancer (80%) [16,17]. Light-microscopic examination of necropsy specimens of periadrenal tissue has shown that the prevalence of BAT is higher in patients with cancer cachexia (80%) than in age-matched controls [3]. Moreover, animal studies have shown that cancer cachexia due to an increased metabolic rate may be mediated by sympathetic stimulation and subsequent BAT thermogenesis with overexpression of UCP1 [18,19]. Cancer patients have very high chronic stress responses caused by psychosocial distress [20,21]. The biological consequences of cumulative stress include catecholamine hyperactivity of the sympathetic nervous system (SNS) response [22], and the SNS plays a key role in the activation of BAT. Our study, which reduced the interference of low outdoor temperature, further demonstrates that more active neoplastic status was associated with more vigorous TMA of BAT.

Our patients with neoplastic status scores of 2 were thought to have either malignancies, inflammatory lesions, or a combination of the two. We hypothesize that inflammation also strongly influences the metabolic activity of BAT in our patients with activated BAT. Inflammatory stimulated pain and stress can activate the fight-or-flight response with subsequent increases in adrenaline and noradrenaline. Lechin et al. [23] reported significant increases in plasma catecholamines in various patient groups during periods of disease exacerbation. The hypothalamic-pituitary-adrenal (HPA) axis and autonomic nervous system are both activated during inflammation as an elaborate multi-directional communication pathway designed to restore homeostasis, in part by regulating the inflammatory and subsequent immune responses. Efferent signals are relayed to the site(s) of inflammation primarily by the secretion of norepinephrine from efferent sympathetic neurons and of epinephrine from the adrenal medulla [24]. On the other hand, tumor necrosis factor alpha (TNF-α, cachectin), a potential mediator of cancer cachexia and the dysregulation of inflammation, increases the thermogenic activity of BAT and the rate of fatty acid synthesis from glucose in BAT assayed *in vitro *[25,26]. These processes provide a reasonable explanation for the association between neoplastic status/inflammation and BAT metabolic activity.

Several factors may affect the activation of BAT. The conversion of white to brown adipocytes can be induced by β-adrenergic stimulation with hormones (catecholamines) that are released in response to stress or low blood sugar [27]. Activated BAT is not present in relevant quantities in adults except during sympathetic stimulation. Physical and chemical stressors, e.g., cold, heat, radiation, noise, vibration, pain, immobilization, and psychological stress, can cause SNS responses [28]. In addition, an *in vivo *animal study [29] reported stress from burn injuries and cutaneous wounds were associated with the activation of ^18^F-FDG accumulation in BAT. BAT activity can be directly inhibited by beta-blockers such as propranolol, and partially reduced by diazepam, the latter probably because anxiety may also activate the SNS [30]. BAT activation has been reported to be more frequent during the cooler seasons of the year; however, the metabolic activity of BAT depends on many factors [13,31]. Consistent with previous studies [9,11-13], our patients with activated BAT were significantly younger, leaner, more often female, had lower fasting blood sugar, and underwent ^18^F-FDG PET/CT during colder outdoor temperature conditions than patients without activated BAT. Although outdoor temperature was related to the prevalence of BAT, none of the outdoor temperature parameters investigated in our study was significantly associated with the TMA of BAT. Few studies have explored the relationship between BAT activity and outdoor temperature. Consistent with our results, Kim et al. [10] found no significant relationship between the intensity of BAT ^18^F-FDG uptake and daily average outdoor temperature. A study [13] done in Quebec, Canada, where the average outdoor temperature is substantially lower than in the tropics, showed that lower outdoor temperature was associated with an increase in the metabolic activity of BAT. We believe that the inconsistent results might be related to how low the outdoor temperature was.

Unlike patients in previous studies done in temperate zones, our patients were in tropical areas. The consistently warm outdoor temperature reduces the stimulating effects of cold environmental temperature on BAT. Univariate and multivariate analyses showed that neoplastic status was the only factor significantly associated with the TMA of activated BAT. We hypothesize that low environmental temperature is an initiator of BAT activation, and neoplastic status is important in the quantified metabolic activity of those activated BAT deposits: it is a promoter. Although we cannot conclude from the data presented that the presence of demonstrable activated BAT is a sign of cancer or the reactivation of cancer, future research on the occurrence of activated BAT and neoplastic status may be warranted.

The current study has several limitations. Because this study was retrospective, indices of the stresses associated with cancer, i.e., measurements of the HPA or SNS activities and other psychological or non-psychological factors that could influence catecholamine levels, were not well assessed. In addition, the number of patients for the analysis of the associations between the TMA of BAT and biological and environmental factors was relatively small. A larger number of patients will be needed for verification.

## Conclusions

In patients with activated BAT, neoplastic status is a critical determinant of BAT activity. A more active neoplastic status was associated with more vigorous BAT metabolic activity.

## List of abbreviations

BAT: brown adipose tissue; ^18^F-FDG: 2-deoxy-2-[18F]fluoro-D-glucose; PET/CT = positron emission tomography/computed tomography; TMA: total metabolic activity; UCP1: uncoupling protein 1; 3D: 3-dimensional; 2D: 2-dimensional; FWHM: full width at half-maximum; PN: posterior neck; SC: supraclavicular; PV: paravertebral; SR: suprarenal; BMI: body mass index; SUV: standardized uptake value; VOI: volume of interest; SE: standard error; SNS: sympathetic nervous system; HPA: hypothalamic-pituitary-adrenal; TNF-α: tumor necrosis factor alpha.

## Competing interests

The authors declare that they have no competing interests.

## Authors' contributions

**YCH: **conception, design, acquiring data, analyzing and interpreting data, drafting the manuscript. **TBC: **analyzing and interpreting data, enhancing its intellectual content. **CCH: **conception, analyzing data, revising the manuscript, enhancing its intellectual content. **SHL, PWW**, **BFL**, **CYK: **interpreting data, enhancing its intellectual content. **NTC: **conception, design, revising the manuscript, enhancing its intellectual content. All authors read and approved the final manuscript.
